# Reconnoitering the Dental Knowledge and Attitude of Pediatricians in the Western Uttar Pradesh Region of India

**DOI:** 10.7759/cureus.60024

**Published:** 2024-05-10

**Authors:** Manisha Kaushik, Avantika Tuli, Shveta Sood

**Affiliations:** 1 Department of Pediatric and Preventive Dentistry, Manav Rachna Dental College, Faridabad, IND

**Keywords:** caries, health, dental, medical professionals, pediatrics

## Abstract

Background

While numerous studies have investigated the prevalence of dental cavities in India, there remains a paucity of research on dental decay across varying age demographics. With early childhood caries (ECC) posing significant threats to young children's holistic health, the role of healthcare practitioners in spreading awareness and preventive measures is crucial. The intention of the present study was to determine pediatricians' opinions on pediatric oral health in the western area of Uttar Pradesh. It also intended to evaluate how these professionals perceived about developing oral health awareness among children, preventing dental caries, and preserving baby oral health.

Methods

About 600 pediatricians (MD) from six regions in western Uttar Pradesh participated in a descriptive cross-section pilot research. To measure dental knowledge as well as attitude, an organized questionnaire was used. Data were then analyzed by applying IBM SPSS Statistics for Windows, Version 21 (Released 2012; IBM Corp., Armonk, New York, United States).

Results

The findings indicate that 56.7% of school-going children nationally experience dental caries, with a concerning 69.1% in the 6-10 age group. ECC prevalence is reported at 49.6% nationally. A significant number of professionals believed in the preventability of dental caries 563 (93.8%) and acknowledged the benefits of routine dental visits 559 (93.2%). Older professionals and those working in hospitals/universities demonstrated higher knowledge and a more positive attitude toward pediatric oral health. Interestingly, there was a robust agreement (545 (90.8%)) among participants that oral health education should be integrated into medical education curricula.

Conclusion

In light of the findings, it's clear that pediatric oral health is an area that demands more focus and awareness, especially in the western region of Uttar Pradesh. While a significant portion of medical professionals show commendable knowledge regarding dental health, there is room for enhancement. Especially striking is the pivotal role a pediatrician can play, given their closeness to families and holistic understanding of a child's health. Utilizing this unrealized potential and encouraging these professionals to integrate dental awareness and practices into their regular encounters are urgently needed. Collaboration with pedodontists may help close the knowledge gap and create a setting where all kids can receive complete dental treatment.

## Introduction

India, with its vast and diverse population, has long been the subject of extensive research when it comes to dental health. Among the myriad of studies conducted, a significant amount of attention has been directed toward understanding the prevalence of dental cavities [1]. However, an observed gap in this body of knowledge is the limited number of investigations that scrutinize dental decay across varying age demographics. In an attempt to bridge this void, a meticulous review was carried out over a span of 24 years, from 1992 to 2016. The findings unveiled that the average incidence rate of caries among school-going children in the nation stood at 56.7% [2,3]. Further dissection of this statistic revealed the prevalence within specific age brackets: 48.9% for those aged 2 to 5, a concerning 69.1% for the 6-10 age group, and 52.1% for adolescents aged 11 to 15 [2,3].

An alarming subset of dental issues that demands immediate attention is early childhood caries (ECC). Characterized by its aggressive nature, ECC specifically targets young children, presenting a formidable challenge to both healthcare professionals and guardians alike. Recent data, as of 2018, by Mehta, showcased a national ECC prevalence rate of 49.6%. Delving deeper into regional variations, the southern state of Andhra Pradesh recorded the highest incidence rate at 63.5%, whereas the northern-eastern state of Sikkim stood at 41.92% [3]. Furthermore, Uttar Pradesh, a key northern state, reported an average occurrence of 49.54% for ECC [4].

The repercussions of ECC are not limited to the mere degradation of dental structures. If left unchecked, ECC has the potential to drastically affect a child's holistic well-being. It paves the way for dental complications, pain, difficulties in daily activities like chewing, potential weight loss, and stunted growth. Moreover, the psychosocial implications include diminished self-esteem and confidence [5].

Given this landscape, the role of healthcare practitioners becomes paramount. Pediatricians often emerge as the front runners in a child's healthcare journey. Their frequent interactions with children, especially during the formative years, position them uniquely to impart crucial knowledge about oral hygiene and preventive care. The American Academy of Pediatric Dentistry (AAPD) recognizes this pivotal role and has been an advocate for establishing a comprehensive "dental home" for children, ideally by their first birthday. This concept seeks to encapsulate a continuous, comprehensive, and coordinated approach toward dental health, which transcends mere treatment and delves deep into anticipatory guidance and developmental understanding [6].

However, an observable lacuna in this framework is the underutilized potential of general practitioners. Their proximity to families, ability to forge long-term relationships, and understanding of a child's overall health dynamics render them invaluable in the domain of pediatric oral health [7].

This research endeavors to navigate through this uncharted territory. By focusing on the western region of Uttar Pradesh - an area with limited existing data - the study aims to elucidate the current understanding, practices, and perspectives of medical professionals about pediatric oral health. Furthermore, it aims to evaluate the attitude of pediatricians regarding the prevention of dental caries, oral health education, and maintaining oral hygiene.

## Materials and methods

In this scientific endeavor, we embarked on an exploratory cross-sectional pilot study aimed at delving into the intricacies of dental knowledge and perspectives within the western regions of Uttar Pradesh. Focusing our attention on six pivotal divisions - Meerut, Moradabad, Bareilly, Agra, Aligarh, and Kanpur - we sought to glean valuable insights. Our sample cohort consisted of 600 individuals, predominantly comprising local pediatricians who held prestigious Doctor of Medicine (MD) degrees. To ensure an optimal recruitment process, we meticulously sourced participants through directories provided by the Uttar Pradesh Indian Academy of Pediatrics. Our sole exclusion criterion pertained to the unwillingness of pediatricians to partake in the study, thereby preserving the integrity of our sample pool.

Ethical considerations formed a cornerstone of our methodology, as evidenced by our acquisition of ethical approval from the Institutional Ethical Committee of Manav Rachna Dental College (IEC/MRDC/2023/021) and the diligent obtaining of consent from all participating individuals. With a commitment to upholding ethical standards, each participant was presented with a comprehensive informed consent form, thereby ensuring a clear understanding and unequivocal agreement to partake in the study. Additionally, to bolster response rates and foster participant engagement, timely reminders were dispatched.

The development of our data collection instrument, a robust questionnaire, underwent rigorous scrutiny by esteemed experts in the field of pediatric dentistry to ensure its validity and relevance. Participants were afforded convenient access to the survey instrument, either through their corporate email accounts or via a Google Docs link accessible on their mobile devices. The questionnaire encompassed various facets, including demographic information, assessment of dental knowledge, and exploration of general professional perspectives.

Statistical analysis

Subsequent to data collection, a meticulous approach was adopted for data analysis. Leveraging the capabilities of IBM SPSS Statistics for Windows, Version 21 (Released 2012; IBM Corp., Armonk, New York, United States), we employed a combination of statistical techniques to discern patterns and derive meaningful insights. Continuous data were meticulously scrutinized, with emphasis placed on standard deviation and mean values for depiction, while categorical data were distilled into concise frequencies for clarity. Furthermore, Microsoft Excel facilitated the creation of visually engaging graphical representations to aid in data interpretation.

To ascertain the statistical significance of our findings, independent and paired t-tests were utilized as baseline parametric significance tests, with a predefined significance level of 0.05. This stringent threshold ensured the robustness of our conclusions and instilled confidence in the validity of our results. Through this meticulously crafted scientific approach, we endeavored to unravel the complexities of dental knowledge and perspectives within the targeted regions of Uttar Pradesh, thereby contributing to the broader body of scientific knowledge in the field of pediatric dentistry.

## Results

A summary of the participants' demographic details and professional traits can be found in Table 1. The sample population is made up primarily of people between the ages of 40 and 55. Over 50% of the participants are married, and there is a fairly even split between the sexes. The bulk of participants work for hospitals or universities, and a sizable percentage have less than 10 years of experience in their fields. Daily, a large percentage of people care for less than 20 patients, while a smaller percentage manage more than 40 patients.

**Table 1:** Demographic characteristics of respondents N: Number; % age: Percentage

Characteristics	Categories	N	% age
Age (years)	25-40	195	32.5%
	40-55	311	51.8%
	55-70	94	15.7%
Gender	Male	291	48.5%
	Female	309	51.5%
Marital status	Married	383	63.8%
	Unmarried	217	36.2%
Practice setting	Private practice	216	36.0%
	Government service	63	10.5%
	Hospital/university	321	53.5%
Work experience	≤10	285	47.5%
	11-15	182	30.3%
	16-20	84	14.0%
	>20	49	8.2%
Number of patients seen per day	<20	235	39.2%
	20-30	173	28.8%
	30-40	114	19.0%
	>40	78	13.0%

With an emphasis on the area of pediatric dentistry, Table 2 gives a thorough summary of how the participants understood and applied oral hygiene practices. A startlingly high percentage of participants, namely 522 (87.00%), showed knowledge of the proper age at which a child should have their first dental appointment. Additionally, a remarkable 498 (83.57%) of respondents correctly identified unsuccessful methods for easing teething discomfort. A significant portion of the respondents, 432 (72.59%), correctly recognized the main cause of dental caries, and an even greater number, 474 (79.05%), properly identified the particular kind of sugar believed to be most cariogenic. A remarkable 510 (85.59%) of participants correctly identified the elements for breastfeeding bottle caries to arise. There are gaps in knowledge regarding the bacteria linked to nursing bottle caries and the crucial function of pH in tooth demineralization, despite the fact that many people are aware of the ideal ages for weaning and the significance of infant dental care. But a huge majority of 588 (98.02%) supported the start of parental participation in kids' oral hygiene practices.

**Table 2:** Distribution of study participants on the basis of positive responses to questions for assessment of their knowledge of oral hygiene n: Number; % age: Percentage

Question	N	% age
At what age child’s first dental visit be scheduled?	522	87.00
Which of the following cannot be used for non-pharmacological management of teething?	498	83.57
Which is more important in causing dental caries?	468	78.57
Most cariogenic sugar	474	79.05
The bacteria responsible for causing nursing bottle caries	414	69.29
Nursing bottle caries result from:	510	85.59
By what age does the American Academy of Pediatrics/American Academy of Pediatric Dentistry (AAPD) recommend that a child be weaned off a bottle?	552	92.05
The main cause of early childhood caries is	432	72.59
Who is responsible for infant oral healthcare	570	95.74
Critical pH at which tooth demineralization occurs:	474	79.76
By what time Parents should begin cleaning a child’s mouth:	588	98.02
At what age should the child start brushing his teeth with fluoridated toothpaste?	390	65.36

The information in Table 3 makes it evident how the participants' knowledge ratings are impacted by their demographic and professional backgrounds. The higher scores received by individuals who are older, especially those between the ages of 55 and 70, demonstrate the significance of age. Significantly, the workplace environment had a significant impact on performance, with workers at hospitals and universities outperforming their colleagues in private practice. Practical experience improved knowledge acquisition, as seen by the better scores received by those with more than 20 years of professional experience. It was demonstrated, however, that factors like gender alongside patient volume had no impact on knowledge levels. The number of daily patient encounters did not correspond with more knowledge, even when men and women scored equally. This claim highlights the nuanced factors that play a role in how one acquires knowledge on a certain topic.

**Table 3:** Comparison between the score of knowledge and demographics of the study participants with the positive response ^: ANOVA; #: Student t-test; * *: Significant

Parameter	Categories	N	Mean ± SD	P-value
Age^^^	25-40	195	9.32 ± 2.67	0.001**
	40-55	311	10.88 ± 1.34	
	55-70	94	11.29 ± 1.19	
Gender^#^	Male	291	10.57 ± 1.19	0.784
	Female	309	10.39 ± 1.17	
Marital status^#^	Married	383	10.84 ± 1.19	0.570
	Unmarried	217	10.21 ± 1.17	
Practice setting^	Private Practice	216	10.05 ± 1.27	0.001**
	Government service	63	10.42 ± 1.48	
	Hospital/University	321	10.98 ± 1.55	
Work experience^	≤10	285	9.55 ± 1.80	0.001**
	11-15	182	10.29 ± 1.64	
	16-20	84	10.87 ± 1.34	
	>20	49	10.94 ± 1.15	
Number of patients^ seen per day	<20	235	10.12 ± 1.16	0.725
	20-30	173	10.39 ± 1.22	
	30-40	114	10.65 ± 1.04	
	> 40	78	10.94 ± 1.26	

Table 4 provides insight into participant's attitudes toward oral hygiene. A resounding 563 (93.8%) believed dental caries is preventable, and 559 (93.2%) thought that routine dental visits can avert oral diseases. About 545 (90.8%) felt that medical curricula should encompass oral health education for children, and 550 (91.7%) believed poor oral health could impede a child's growth and development. Incorporating basic dental checkups into pediatricians' daily routines was supported by 483 (80.5%). Furthermore, 491 (81.8%) acknowledged the importance of disseminating information about dental caries prevention and infant oral healthcare for better parent counseling. A notable 552 (92%) agreed on the significance of nutrition and oral hygiene for children's dental health. Concerning the behavior's impact on tooth decay, 453 (75.5%) concurred that children's or parents' oral hygiene habits could heighten risks. Last, collaboration with pedodontists for improved child oral health was endorsed by 532 (88.7%).

**Table 4:** Distribution of the study participants on the basis of positive response to questions for assessment of their attitude towards oral hygiene. N: Number; %: Percentage

Question	Number/percentage	Agree	Disagree	Indecisive
Do you agree dental caries can be prevented?	N	563	3	34
	%	93.8	0.5	5.67
Do you agree oral disease can be prevented by routine dental visit?	N	559	4	37
	%	93.2	0.7	6.2
Do you agree oral health education should be included in the medical education curriculum for preventing oral diseases in children?	N	545	16	39
	%	90.8	2.7	6.5
Do you agree poor oral health can cause lack of proper growth and development in children?	N	550	18	32
	%	91.7	3.0	5.3
Do you agree to include basic dental checkup activities in pediatricians daily practice?	N	483	64	53
	%	80.5	10.7	8.8
Do you agree providing availed information about the prevention of dental caries, and infant oral health care help the medical practitioners’ to counsel parents for better oral health	N	491	53	66
	%	81.8	8.8	9.3
Do you agree proper nutrition, maintaining oral hygiene is important for overall dental health in the children	N	552	22	26
	%	92.0	3.7	4.3
Do you agree the behavior of child/parent’s regarding oral hygiene the increase the risk of tooth decay?	N	453	45	102
	%	75.5	7.5	17.0
Do you agree collaboration with pedodontists can perform better care regarding the infant oral health care and prevention of dental caries in children?	N	532	11	47
	%	88.7	1.8	9.5

The results in Table 5 indicate that the pediatrician's positive attitude toward oral health is significantly influenced by age (p-value = 0.001), with mean scores increasing from 7.67 ± 2.67 in the 25-40 age group to 8.39 ± 1.19 in the 55-70 age group. Gender and marital status did not significantly affect attitude (p-values 0.824 and 0.614, respectively), with mean scores of 8.03 ± 1.19 for males, 8.07 ± 1.17 for females, 7.99 ± 1.39 for married, and 8.09 ± 1.26 for unmarried. Practice setting was significant (p-value = 0.001), with mean scores of 10.79 ± 1.55 for hospital/university settings compared to 9.37 ± 1.27 in private practice and 9.95 ± 1.48 in government service. Work experience also significantly influenced attitudes (p-value = 0.001), with mean scores ranging from 7.14 ± 1.22 in those with ≤10 years of experience to 8.24 ± 1.08 in those with >20 years. Number of patients seen per day was not significant (p-value = 0.637), with mean scores ranging from 7.89 ± 1.19 to 8.13 ± 1.26.

**Table 5:** Different demographic parameters affecting the pediatrician's positive attitude toward oral health **: Significant

Parameter	Categories	N	Mean ± SD	P-value
Age	25-40	195	7.67 ± 2.67	0.001**
	40-55	311	8.02 ± 1.34	
	55-70	94	8.39 ± 1.19	
Gender	Male	291	8.03 ± 1.19	0.824
	Female	309	8.07 ± 1.17	
Marital status	Married	383	7.99 ± 1.39	0.614
	Unmarried	217	8.09 ± 1.26	
Practice setting	Private practice	321	7.31 ± 1.27	0.001**
	Government service	285	7.95 ± 1.01	
	Hospital/university	182	8.36 ± 1.08	
Work experience	≤10	84	7.14 ± 1.22	0.001**
	11-15	49	7.42 ± 1.08	
	16-20	235	7.89 ± 1.00	
	>20	173	8.24 ± 1.08	
Number of patients seen per day	<20	114	7.89 ± 1.19	0.637
	20-30	78	7.95 ± 1.52	
	30-40	195	8.01 ± 1.06	
	>40	311	8.13 ± 1.26	

## Discussion

This cross-sectional pilot study of medical professionals in western Uttar Pradesh, India, gave a clear picture of pediatrician demographics, knowledge of oral health, and attitude. A total of 600 pediatricians were included as participants in the pilot trial. The demographic information showed that the majority of those surveyed, 311 (51.8%), were between the ages of 40 and 55, indicating a sizable proportion of professional caregivers. The gender ratio was about equal, with slightly more women, 309 (51.5%), than men, 291 (48.5%). A total of 383 (63.8%) of those surveyed said they were married. More people (53.5%) worked in the hospital or university setting, and 285 (47.5%) had less than 10 years of experience in their field. The majority of doctors saw little more than 20 people every day. Our investigation uncovered an encouraging pattern: a significant proportion of practitioners had deep expertise in critical facets of child dental treatment. These topics included the ideal age for a child's first dental appointment, safe teething remedies, the underlying causes of dental caries, and a dedication to adhering to the AAPD/American Academy recommendations.

The study involved the observation of responses to the inquiry on the appropriate age at which a child's initial dental appointment ought to be arranged. Approximately 552 (87%) were aware of the appropriate age for a child's initial dental visit. This is significant as early visits enable timely interventions for oral health. It is advisable to schedule dental appointments at an early age as it promotes proactive measures, timely identification of oral health issues, guidance on appropriate dietary choices and nutritional practices, instructions on maintaining oral hygiene, and avoidance of non-nutritive sucking behaviors [8]. The AAPD recommends that children should undergo their initial dental examination no later than six months after birth, or promptly after the eruption of their first tooth, and no sooner than 12 months of age. A variety of behaviors are categorized as oral habits, including pacifier and finger sucking, lip and nail-biting, bruxism, self-harming behaviors, mouth breathing, and tongue push. A total of 468 (78.57%) participants correctly recognized poor oral hygiene as the main contributor to dental caries, which is a considerable percentage.

Additionally, a sizable number of 474 (79.5%) pediatricians offered dietary recommendations about items. In contrast, according to Sabbagh et al.'s results, just 30% of Saudi Arabians followed suit. Regularly eating snacks that are high in carbs in between mealtimes has been linked to a higher risk of developing dental caries, especially in people who have poor oral clearance rates. As a result, it is advised to have highly acidogenic sugar-containing foods between meals to decrease the risk of acquiring dental caries. Instead, snacks prior to meals should include hypo- or non-acidogenic choices [9]. Almost 69.29% could correctly identify the bacteria, *Streptococcus mutans*, responsible for nursing bottle caries. Knowledge regarding the transfer of *S. mutans*, primarily from maternal caregivers, seems vital. *S. mutans* has the potential to be transferred through two primary modes: vertical transmission, which occurs from mother to child, and horizontal transmission, which takes place among individuals within a group, such as relatives or classmates in a classroom. There was a substantial association observed between the colonization of *S. mutans* and various factors, including maternal oral hygiene, eating habits, child-rearing behaviors, sharing of food and utensils, as well as breastfeeding and co-sleeping with the mother. The primary origin from which infants receive *S. mutans* is their maternal caregivers [10,11].

A majority of 552 (92.05%) knew the recommended age by AAP and AAPD for children to stop using bottles. Several feeding practices, such as night breastfeeding, on-demand breastfeeding, and prolonged weaning past the age of two years, have the potential to negatively impact dental health [12,13]. In their study, Sabbagh et al. discovered that a significant proportion of pediatricians (81.3%) demonstrated familiarity with the detrimental consequences associated with night bottle feeding in children [9]. In a prior study conducted by Murthy and Mohandas, it was found that a majority of clinicians, above 50%, had the belief that ECC exclusively affects infants who are fed by bottle feeding. However, empirical research suggests that infants who co-sleep with their mothers and engage in nocturnal breastfeeding exhibit a heightened susceptibility to dental caries [14]. In addition, the study examined the level of awareness among medical practitioners regarding their responsibilities in newborn oral health care. An overwhelming number of 570 (95.74%) recognized their essential role in providing dental health care advice to parents. Less than half, 474 (79.76%), correctly identified the critical pH level at which tooth demineralization occurs, suggesting a need for more education in this area.

According to the AAP, fluoride toothpaste should be introduced as soon as the first tooth appears. However, 390 (65.36%) participants knew the right age to start using fluoride toothpaste. In a recent survey, a significant majority of professionals (92.86%) were found to possess an outdated understanding regarding the usage of fluoride, which may potentially lead to inappropriate results [15]. On the other hand, Al-Hussyeen et al. noted in their study that a significant proportion of pediatricians did not consistently incorporate dentition assessments as part of their usual examinations [16].

The majority (over 90%) of the participants believe in the preventive aspects of dental health, as evidenced by their belief that dental caries can be avoided (93.8%) and that routine dental visits can prevent oral diseases (559 (93.2%)). This demonstrates a strong awareness and acceptance of the fundamental principles of dental health prevention. There is also a strong inclination, 548 (90.8%), among the participants that oral health education should be a part of the medical education curriculum, underscoring the recognition of the significance of early education in promoting oral health. The *Journal of the Indian Society of Pedodontics and Preventive Dentistry* advocates for the routine incorporation of dental care assessment [17]. In light of the high incidence of dental issues in children, it is crucial for pediatricians to integrate oral health into their regular clinical protocols. A pediatrician with a thorough knowledge of common dental diseases, risk evaluation, implementation of preventive strategies, and collaboration with dental practitioners can make a valuable contribution to the oral health and general welfare of children [18].

The idea of integrating dental checkups into the daily routines of pediatricians saw slightly reduced support, with 483 (80.5%) in agreement. This suggests that while oral health is recognized as essential, its integration into broader medical practice is still a topic of debate. The link between oral health and overall growth and development in children is firmly established, with 550 (91.7%) agreeing that poor oral health can stunt proper growth and development. A complete understanding of the association between dental caries and nutrition is vital for pediatricians, considering their crucial role in offering nutritional counseling. Having a comprehensive understanding of this information is crucial for healthcare professionals to successfully educate their patients on the adverse impacts of cariogenic diets. Insufficient adherence to oral hygiene practices might result in various adverse outcomes, encompassing bodily discomfort, mental misery, reduced self-assurance, and a compromised capacity to do everyday activities. Children with suboptimal dental health may encounter impaired growth, insufficient weight gain, heightened irritability, enhanced vulnerability to illnesses, elevated hospitalization rates, and disturbed sleep habits [19].

Providing information to medical practitioners about dental caries and infant oral health care is considered beneficial by 491 (81.8%) respondents, indicating a consensus on the value of interdisciplinary knowledge sharing. The importance of proper nutrition and maintaining oral hygiene for overall dental health in children is nearly universally accepted by 552 (92%) participants in agreement. There is a symbiotic relationship between dental health and nutrition. Oral infectious diseases, in addition to acute, chronic, and terminal systemic conditions that exhibit oral signs, exert a substantial influence on an individual's ability to engage in food consumption as well as their dietary and nutritional status. Similarly, the influence of dietary habits and nutrition on the development and upkeep of the oral cavity, as well as the progression of oral disorders, is substantial [20]. These elements play a significant role in the intricate etiology and pathophysiology of oro-facial illnesses and disorders. The conduct demonstrated by parents possesses the capacity to affect their approach to parenting, thereby exerting an impact on the maturation and advancement of their offspring. Over the initial years of growth, children often adopt behavioral patterns from their elders. This process is primarily influenced by parental attention toward the child's needs and emotions, as well as the parent's ability to convey the consequences associated with both positive and negative acts [21].

When discussing the behavior of a child or their parents concerning oral hygiene, there is a sizable proportion (17.0%) who remain indecisive about whether it directly increases the risk of tooth decay. However, the majority, 453 (75.5%), still agree with the correlation. Last, collaboration with pedodontists was seen favorably by 532 (88.7%) participants, suggesting that interdisciplinary collaboration is recognized as a potential means to enhance care related to infant oral health and prevent dental caries in children. A statistically significant association exists between age and a pediatrician's perspective on oral health, as evidenced by a p-value of 0.001. As the age of individuals increases, there is a discernible increase in the average scores observed between the age groups of 25-40 (mean score of 7.67) and 55-70 (mean score of 8.39). This observation implies that older pediatricians may possess greater familiarity and expertise in the realm of oral health concerns.

Gender doesn't significantly influence a pediatrician's attitude toward oral health (p-value = 0.459). The mean scores for males (8.03) and females (8.07) are relatively close; marital status is not a significant factor influencing a pediatrician's attitude (p-value = 0.557). Married pediatricians have a slightly higher mean score (9.72) than unmarried ones (9.44). The setting in which a pediatrician practices also has a significant impact on their attitude (p-value = 0.001). Pediatricians working in hospitals or universities (mean score of 10.79) seem to have a more positive attitude than those in private practice (9.37) or government service (9.95). This could be because those in hospital or university settings are more exposed to academic discussions, research, or diverse patient profiles, emphasizing the importance of oral health.

The number of years of experience a pediatrician has significantly shaped their attitude toward oral health (p-value = 0.001). There is a trend indicating that as pediatricians gather more experience, their positive attitude toward oral health seems to increase. Those with over 20 years of experience have the highest mean score of 10.34. This might be due to accumulated knowledge, exposure to a variety of cases, or perhaps a growing understanding of the interconnectedness of oral health with overall health over time.

The number of patients a pediatrician sees daily doesn't significantly affect their attitude (p-value = 0.637). Whether a pediatrician sees fewer than 20 patients (mean score of 9.02) or more than 40 (mean score of 9.46), their attitude toward oral health remains relatively constant. This implies that patient volume doesn't necessarily correlate with their perspective on the importance of oral health.

This study may be limited by factors such as potentially non-representative sample size or sampling methods, reliance on subjective data collection methods prone to biases, the cross-sectional design's inability to capture longitudinal trends, language and cultural barriers impacting data validity, resource constraints affecting the depth of the study, contextual factors unique to Western Uttar Pradesh influencing generalizability, and the potential for various biases throughout the research process.

## Conclusions

This comprehensive cross-sectional pilot study offers invaluable insights into the knowledge and attitude of medical practitioners in the western region of Uttar Pradesh, India, concerning oral health in pediatric patients. The data underscores a commendable understanding and positive attitude toward the preventive aspects of dental health among participants. Yet, it also pinpoints areas, like knowledge about critical pH levels for tooth demineralization, where continued education is imperative. Demographic factors such as age, workplace setting, and years of professional experience notably influence a pediatrician's disposition toward oral health. This study underscores the importance of an interdisciplinary approach in pediatric health care and the need to continually update and integrate oral health knowledge into the broader medical curriculum and practice.
